# Therapeutic Potential of Vitamin B Complex in Peripheral Nerve Injury Recovery: An Experimental Rat Model Study

**DOI:** 10.3390/medicina60091556

**Published:** 2024-09-23

**Authors:** Ahmet Kahraman, Metin Temel, Numan Atilgan, Ahmet Saray, Recep Dokuyucu

**Affiliations:** 1 Department of Plastic Reconstructive and Aesthetic Surgery, Mustafa Kemal University, Hatay 31230, Turkey; prcahmet@yahoo.com.tr (A.K.); prsdrmetintemel@gmail.com (M.T.); 2 Department of Hand Surgery, Private Clinic, Gaziantep 27060, Turkey; doktor_dao@hotmail.com; 3 Department of Orthopedics and Traumatology, Private Deva Hospital, Gaziantep 27060, Turkey; a.asaray40@gmail.com; 4 Department of Physiology, Medical Specialization Training Center (TUSMER), Ankara 06230, Turkey

**Keywords:** peripheral nerve injury, nerve healing, vitamin B complex, rat

## Abstract

*Objectives*: Vitamin B complexes are frequently used in clinical practice for peripheral nerve trauma. However, there is a lack of scientific data on their effectiveness. This study aims to investigate the impact of the vitamin B complex on nerve recovery in a rat model of peripheral nerve paralysis. *Materials and Methods*: Sixty male Wistar Albino rats were divided into six groups. Models of nerve injury, including blunt trauma, nerve incision, and autograft, were performed on all rats approximately 1 cm distal to the sciatic notch. B-complex vitamins were injected intraperitoneally at 0.2 mL/day to the treatment groups. The control groups were given 0.2 mL/day saline. After 1 month, the study was terminated, electromyography (EMG) was performed to measure the conduction velocity, and nerve tissue was taken from the repair line. The sciatic function indexes (SFIs) were calculated and analyzed. The histopathological samples were stained with hematoxylin and eosin and Toluidine blue and examined with a light microscope. Pathologically, myelination, fibrosis, edema, and mast cell densities in the nervous tissue were evaluated. *Results*: The vitamin B treatment groups demonstrated significant improvements in SFI compared to the control groups, indicating functional improvement in nerve damage (*p* < 0.05). In the nerve graft group, the vitamin B group showed a shorter latency, higher velocity, and larger peak-to-peak compared to the controls (*p* < 0.05). In the nerve transection group, the vitamin B group had better latency, velocity, and peak-to-peak values than the controls (*p* < 0.05). In the crush injury group, the vitamin B group exhibited an improved latency, velocity, and peak-to-peak compared to the controls (*p* < 0.05). Better myelination, less fibrosis, edema, and mast cells were also in the vitamin B group (*p* < 0.05). *Conclusions*: Vitamin B treatment significantly improves nerve healing and function in peripheral nerve injuries. It enhances nerve conduction, reduces fibrosis, and promotes myelination, indicating its therapeutic potential in nerve regeneration.

## 1. Introduction

Vascular permeability increases after trauma in the peripheral nerve. Blood flow decreases due to local edema, which is caused by exudative fluid accumulation around the nerve. As a result, ischemia and Wallerian degeneration develop [1]. Corticosteroids suppress this process with their anti-inflammatory effects [2]. Oxidative stress and reactive oxygen species (ROS), generated from free fatty acids in cell membranes, are thought to significantly contribute to neuronal cell death following trauma. Antioxidants help prevent cell death by inhibiting lipid peroxidation and reducing the production of ROS [3].

Treatment of peripheral nerve injuries is one of the most important challenges we encounter in clinical practice [4]. Today, developments in molecular biology and microsurgical techniques have greatly improved peripheral nerve repair procedures [4]. Animal model studies, especially in the last 10 years, have led to significant advances in the contribution of the treatment of peripheral nerve injury to histological and functional recovery [5,6]. However, adequate recovery results have not yet been achieved in the treatment of peripheral nerve damage in humans [7]. The main reason for this difference in the results between animal experiments and clinical experiments is due to the longer distance between the damage points in the target organ and peripheral nerve in humans [8,9]. In addition, the nerve regeneration rate is relatively slow compared to other tissues, and it may take up to 3 months or even 10 months for axons to reach the target organs and tissues they need to reach. Therefore, the treatment must be used in patients for a long time [10].

Neurotropic substances such as nerve growth factor, brain-derived neurotrophic factor, and neurotrophins have an important place in nerve regeneration. Only a few of these factors can be used in clinical practice due to their side effects and very high costs [6,11]. Therefore, it has become necessary to investigate and find other factors for peripheral nerve regeneration [4].

Recently, non-invasive imaging modalities have become increasingly valuable in evaluating peripheral nerve integrity. High-field magnetic resonance imaging (MRI) enables a detailed visualization of the peripheral nerves and their fascicles, allowing for a precise assessment of nerve architecture. Similarly, tomographic high-resolution ultrasound has been shown to effectively depict the nerve fascicles and surrounding tissues, improving diagnostic accuracy [12]. Micro-CT, although less commonly used, has also been applied in preclinical studies to depict peripheral nerve structures with high spatial resolution. Advanced MRI sequences such as the three-dimensional pulsed gradient spin-echo can even image water molecule diffusion within nerve fascicles, a crucial process for assessing nerve integrity [13]. These radiological techniques provide comprehensive insights into the peripheral nerve structure and regeneration, which can complement traditional histological assessments in both experimental and clinical settings. Incorporating these methods into research and clinical practice could improve the diagnostic and prognostic evaluation of nerve injuries, thus enhancing treatment outcomes.

Vitamin B is water-soluble and cannot be stored in the body. Deficiencies in vitamins like thiamine, pyridoxine, riboflavin, and cyanocobalamin can lead to conditions such as convulsions, carpal tunnel syndrome, and chronic pain [14]. Research indicates that the vitamin B complex benefits various pathological conditions, especially painful diseases, even without deficiency [11]. Pyridoxine and thiamine are used to alleviate pain from neuropathic diseases, carpal tunnel syndrome, and premenstrual tension [15]. Vitamin B complexes are also commonly applied in peripheral nerve trauma treatment.

This study aims to evaluate the effect of vitamin B complex supplementation on the histological and functional recovery of peripheral nerves in a rat model of induced nerve paralysis, hypothesizing that the vitamin B complex promotes nerve regeneration and improves recovery outcomes.

## 2. Materials and Methods

### 2.1. Animals and Experimental Procedures

In determining the number of subjects in the group, the decision was made by taking into account the statistical interpretation and significance of the results and the possibility of animal loss during the experiments. In determining the groups, previous studies in the literature were taken into consideration [16,17]. The animals used in the experiment were male Wistar Albino rats aged 12–16 weeks with an average weight of 250–300 g. All animals were housed in the same controlled environment with a consistent temperature, humidity, and 12 h light/dark cycles. The rats were fed the same standard laboratory diet and had access to water ad libitum throughout the study.

### 2.2. Surgical Applications

**Figure 1:**
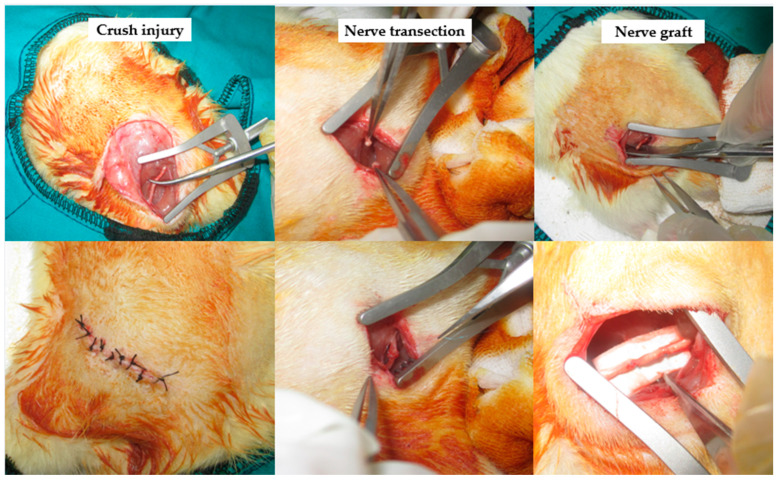
Surgical procedures according to groups.

The surgical procedure was applied to all rats in the same manner. Xylazine (Rompun^®^, Bayer, Germany) was administered intraperitoneally at a dose of 10 mg/kg, and ketamine (Ketalar^®^, Pfizer, New York, NY, USA) was administered intraperitoneally at a dose of 10 mg/kg. After the reflexes disappeared, the surgery was performed in the following standard order. An approximately 15 mm linear incision was made on the dorsal surface of the rat’s right thigh. Blunt dissection continued from the subcutaneous plane, and the sciatic nerve was exposed between the femoral biceps and semi-tendinous muscles using 12 cm hemostatic tweezers. The following procedures were performed according to groups, approximately 1 cm distal to the sciatic notch [16,17] (Figure 1).

### 2.3. Groups

Control group of group 1 (crush injury group control): For crush injury, a linear incision of approximately 15 mm was made on the dorsal surface of the animal’s right thigh. Blunt dissection was continued from the subcutaneous plane, and the sciatic nerve was exposed between the femoral biceps and semitendinosus muscles using 12 cm hemostatic tweezers. The right sciatic nerve was compressed by means of a clamp for 2 min, and a crush injury was created [16,17]. The skin and subcutaneous tissue were closed properly, and the rat was awakened. These rats were fed with rat food and water without restriction throughout the study. Rats in this group were given physiological saline via oral gavage for 1 month.

Group 1 (crush injury to the sciatic nerve + vitamin B complex): The right sciatic nerve was subjected to compression using clamps for 2 min, and a crush injury was created. The skin and subcutaneous tissue were closed properly, and the rat was awakened. The vitamin B complex was given to the animals via oral gavage for 1 month. Each tablet of B-complex vitamin was dissolved in 5 mL of saline and given once a day at a dose of 0.2 mL per rat. The vitamin B complex contained 25 mg of thiamin hydrochloride (Vitamin B1), 2.734 mg of riboflavin phosphate (Vitamin B2), 5 mg of pyridoxine (Vitamin B6), 15 mcg of vitamin B12, 50 mg of niacinamide, and 17.2 mg of D-Panthenol in every 2 mL (Bemiks^®^, Zentiva, Istanbul, Turkey).

The control group of group 2 (sciatic nerve transection group): For the sciatic nerve incision, a linear incision of approximately 15 mm was made on the dorsal surface of the animal’s right thigh. Blunt dissection was continued from the subcutaneous plane, and the sciatic nerve was exposed between the femoral biceps and semitendinosus muscles using 12 cm hemostatic tweezers. The right sciatic nerve was cut with a scalpel, and an end-to-end anastomosis of the nerve ends was performed [16,17]. The skin and subcutaneous tissue were closed properly, and the rat was awakened. These rats were fed with rat food and water without restriction throughout the study. The rats in this group were given physiological saline via oral gavage for 1 month.

Group 2 (sciatic nerve transection + B vitamin complex): The right sciatic nerve was cut with a size 15 sharp scalpel, and the nerve endings were immediately anastomosed end-to-end with a 9/0 prolene suture. The skin and subcutaneous tissue were closed properly, and the rat was awakened. The rats in this group were given the vitamin B complex via oral gavage for 1 month.

Control group of group 3 (nerve graft control group): For sciatic nerve graft repair, a linear incision of approximately 15 mm was made on the dorsal surface of the animal’s right thigh. Blunt dissection was continued from the subcutaneous plane, and the sciatic nerve was exposed between the femoral biceps and semitendinosus muscles using 12 cm hemostatic tweezers. The right sciatic nerve was cut from two separate places with a scalpel, and the nerve region that was released in the middle was sutured back to both ends as an autograft, preserving the position it was cut in [16,17]. The skin and subcutaneous tissue were closed properly, and the rat was awakened. The rats in this group were given physiological saline via oral gavage for 1 month.

Group 3 (sciatic nerve graft repair + B vitamin complex): The right sciatic nerve was made by making two separate incisions: 1 cm distal to the sciatic notch and 1 cm distal to the first incision, with a sharp No. 15 scalpel. The nerve area released in the middle was sutured again with a 9/0 prolene suture as an autograft, preserving the position in which it was cut at both ends. The skin and subcutaneous tissue were closed properly, and the rat was awakened. The rats in this group were given the vitamin B complex via oral gavage for 1 month.

### 2.4. Electrophysiological Evaluation

This study was terminated 1 month after the surgical procedure. EMG was performed to measure the nerve recovery and conduction velocity. EMG was performed at room temperature with a Dantec Key Point EMG device. During EMG, the stimulation electrode was placed distal to the sciatic notch and the recording electrode was placed on the gastrocnemius muscle, 1 cm distal to the tibial tubercle. There was a 3–4.5 cm gap between the anode and cathode. The amplifier frequency limit was taken as 500 hz–10 khz. Each potential was recorded 5 times. A latency measurement was made according to the point where the potential first separated from the isoelectric line. Amplitudes were measured from peak-to-peak and these values were compared with the healthy side. After EMG, the rats were sacrificed, and a 2 cm nerve segment that was 1 cm distal to the sciatic notch was taken for histopathological examination.

### 2.5. Histopathological Evaluation

A marking suture was placed on the proximal part where the biopsy would be taken, including 0.5 cm proximal and distal of the repair line. Each sample was numbered and sent to the pathologist, and since the pathologist did not know which group the sample belonged to, a blind evaluation was made.

For the histopathological examination, it was fixed in 10% formaldehyde solution for 24 h. The proximal, repair line and distal parts of the nerve tissue were stained with tissue samples in different colors. After routine tissue tracing and paraffin embedding, 4-micrometer cross-sections were taken from the paraffin blocks. Sections were stained with hematoxylin and eosin and Toluidine blue [18]. An Olympus BH-2 (Olympus CX31RTSF Olympus Optical, Tokyo, Japan) was used.

### 2.6. Walk Test Analysis

Motor function was assessed by observing the free-walking patterns, a method initially described by De Medinaceli [19]. Twelve weeks after the left nerve surgery, a walking test analysis was performed. The parameters measured included the print length (PL), which is the distance from the heel to the toe; toe spread (TS), the distance between the first and fifth toes; and intermediary toe spread (ITS), the distance from the second to the fourth toes. Each animal’s sciatic functional index (SFI) was calculated using the formula developed by Bain et al. [20]. The SFI is a metric for dysfunction and is presented as a percentage. A SFI of −100% denotes a complete sciatic nerve lesion, while SFI values between −10% and +10% indicate normal function [21,22,23].

### 2.7. Statistical Analysis

All statistical analyses were performed using SPSS software (version 27.0, IBM Corporation, Armonk, NY, USA). The Shapiro–Wilk test was conducted to assess the normality of the data. The results are presented as the mean ± standard deviation (SD). Statistical analysis was performed to determine the significance of the differences between the control and vitamin B treatment groups. The Kruskal–Wallis test was used to analyze the sciatic function indexes (SFIs) between the groups, as it is a non-parametric test suitable for comparing more than two independent groups. A *p*-value of less than 0.05 was considered statistically significant. For the electrophysiological evaluation, the latency, velocity, and peak-to-peak values obtained from electromyography (EMG) were compared between the control and treatment groups using the Student’s *t*-test. This parametric test was chosen because the data were normally distributed, as confirmed by the Shapiro–Wilk test. The histopathological results, including the myelination, fibrosis, edema, and mast cell densities, were also analyzed using the Student’s *t*-test to compare the differences between the control and vitamin B treatment groups.

## 3. Results

### 3.1. Walk Test Analysis

**Figure 2:**
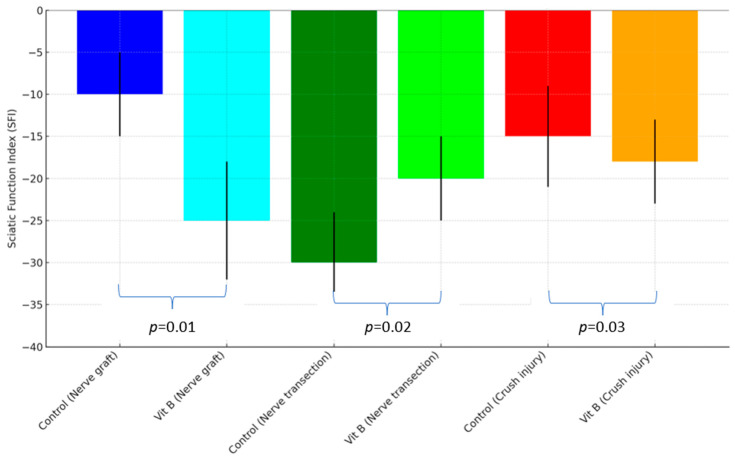
Comparison of sciatic function index (SFI) between groups.

There was a statistically significant difference between the control and treatment groups (*p* < 0.05). Significant improvements were observed in the vitamin B treatment groups compared to the control groups. These results show that vitamin B provides a functional improvement in nerve damage, and its therapeutic effect is statistically significant (*p* < 0.05) (Figure 2).

### 3.2. Electrophysiological Evaluation

A comparison of the electromyography (EMG) results of the groups is shown in Table 1. For the control group with nerve grafts, the latency is 3.5 ± 0.4 ms, the velocity is 45 ± 5 m/s, and the peak-to-peak value is 0.8 ± 0.1 millivolts. In the vitamin B group with nerve grafts, the latency is 3.0 ± 0.3 ms, the velocity is 55 ± 6 m/s, and the peak-to-peak value is 1.2 ± 0.1 millivolts. In the nerve graft group receiving the vitamin B treatment, a statistically significant shorter latency (*p* = 0.011), higher velocity (*p* = 0.035), and larger peak-to-peak (*p* = 0.032) were observed compared to the control group. These results indicate that vitamin B improves nerve conduction velocity and nerve function.

For the control group with a nerve transection, the latency is 4.0 ± 0.5 ms, the velocity is 40 ± 4 m/s, and the peak-to-peak value is 0.5 ± 0.1 millivolts. In the vitamin B group with a nerve transection, the latency is 3.3 ± 0.4 ms, the velocity is 50 ± 5 m/s, and the peak-to-peak value is 1.0 ± 0.2 millivolts. Similarly, in the nerve transection group, the group receiving the vitamin B treatment exhibited a shorter latency (*p* = 0.027), higher velocity (*p* = 0.045), and larger peak-to-peak (*p* = 0.023) compared to the control group. This demonstrates the supportive effect of vitamin B on nerve healing.

For the control group with a crush injury, the latency is 4.2 ± 0.6 ms, the velocity is 38 ± 4 m/s, and the peak-to-peak value is 0.6 ± 0.1 millivolts. In the vitamin B group with a crush injury, the latency is 3.6 ± 0.4 ms, the velocity is 48 ± 5 m/s, and the peak-to-peak value is 1.1 ± 0.2 millivolts. The effects of vitamin B are also evident in the crush injury group. The treated group showed better latency (*p* = 0.015), higher velocity (*p* = 0.038), and a larger peak-to-peak (*p* = 0.035). This indicates that vitamin B contributes to nerve regeneration and healing (Table 1).

### 3.3. Histopathological Evaluation

**Figure 3:**
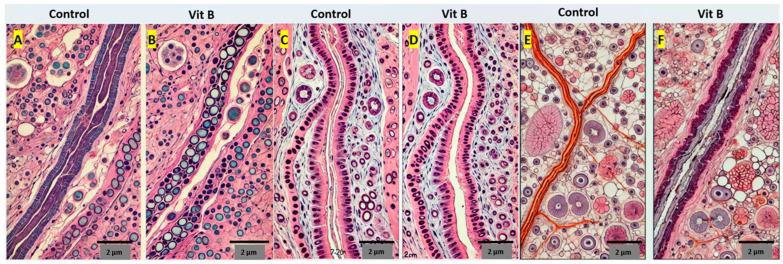
(**A**) In transverse sections stained with Giemsa and Mason, the connective tissue sheaths of the nerve appeared in their normal structure. (**B**) Minimal edema was evaluated between the axons as vacuolization in the endoneurium layer. Sections stained with phosphotungstic acid-hematoxylin (PTAH) displayed irregularities and variations in the thicknesses of the myelin sheaths, along with non-myelinated axons wrapped in Schwann cell sheaths among the myelinated axons. (**C**) In other transverse sections stained with Giemsa and Mason, dense fibrosis and small axons were observed around the nerve’s peripheral region, whereas medium-sized axons were detected in the central region without fibrosis. (**D**) Sections stained with PTAH showed that thin to medium-thick myelin sheaths were predominant, with occasional irregularities and fluctuations. Minimal fibrosis was found in the endoneural tissue between the axons, and unmyelinated axons were also present. (**E**) Additional sections stained with Giemsa and Mason revealed varying sizes of axon sections accompanied by minimal edema. (**F**) In sections stained with PTAH, medium-sized myelinated axons with regular myelin sheaths were primarily observed. Some myelinated axons exhibited vacuoles between the axolemma and myelin sheaths, and there were also partially [12] myelin-free axon sections.

A comparison of the histopathological results between groups is shown in Table 2. In the nerve graft group receiving the vitamin B treatment, higher myelination (*p* = 0.010), lower fibrosis (*p* = 0.030), lower edema (*p* = 0.023), and lower mast cells (*p* = 0.036) were observed compared to the control group. This indicates that vitamin B supports nerve regeneration and healing (Table 2, Figure 3A,B).

In the nerve transection group, the group receiving the vitamin B treatment exhibited higher myelination (*p* = 0.022), lower fibrosis (*p* = 0.041), lower edema (*p* = 0.034), and lower mast cells (*p* = 0.045) compared to the control group. These results demonstrate the supportive effect of vitamin B on nerve healing (Table 2, Figure 3C,D).

In the crush injury group, the group receiving the vitamin B treatment showed higher myelination (*p* = 0.013), lower fibrosis (*p* = 0.034), lower edema (*p* = 0.027), and lower mast cells (*p* = 0.038) compared to the control group. This indicates that vitamin B contributes to nerve regeneration and healing (Table 2, Figure 3E,F).

## 4. Discussion

Our study demonstrated that the vitamin B complex significantly improves the sciatic function indexes (SFIs), electrophysiological parameters, and histopathological outcomes in the treated groups compared to the controls. These results suggest that the vitamin B complex positively impacts nerve regeneration and functional recovery in various models of nerve injury, including nerve grafts, nerve transections, and crush injury.

The histological assessments in our study revealed that the vitamin B complex treatment significantly enhances myelination and reduces fibrosis, edema, and mast cell infiltration across all injury models. These findings are in agreement with the results of Ehmedah et al. (2019), who observed similar improvements in myelination and reductions in fibrosis and inflammation with vitamin B treatment [24]. Our study further supports these conclusions by providing detailed quantitative assessments of the improvements in these parameters. The reduction in fibrosis is particularly notable, as fibrosis is a common obstacle to nerve regeneration. Treatments like methylprednisolone, while effective at reducing inflammation, are associated with increased scar formation and delayed healing [25]. In contrast, the vitamin B complex not only reduces inflammation but also minimizes fibrosis, which may contribute to a more effective long-term recovery.

In recent years, non-invasive imaging modalities, such as high-field MRI and high-resolution ultrasound, have been increasingly utilized to assess peripheral nerve injuries. These techniques allow for the detailed visualization of nerve fascicles and the surrounding tissue architecture. Studies have demonstrated that these advanced imaging techniques are valuable in assessing nerve integrity and regeneration in both clinical and experimental settings [12,13]. While our study focused on histological evaluation, integrating these radiological modalities could provide an even more comprehensive assessment of nerve recovery, as suggested by Pusnik et al. (2024). Combining radiological and histological findings would allow for a more holistic understanding of nerve regeneration dynamics.

Peripheral nerve injuries (PNIs) represent a significant clinical challenge due to the complexity of the regenerative process and the need for effective therapeutic interventions. While vitamin B complexes have shown promise in enhancing nerve recovery, other pharmacological agents and therapeutic strategies are also being explored. Methylprednisolone, a corticosteroid, is often used to reduce inflammation and prevent secondary damage after nerve injury. Studies have shown that it can improve functional outcomes and reduce scar formation, but it may also have side effects such as immunosuppression and delayed wound healing [25,26,27]. Compared to methylprednisolone, the vitamin B complex appears to offer benefits without the associated risks of corticosteroids. Gabapentin is widely used for neuropathic pain management following nerve injuries. It has been shown to promote functional recovery and reduce pain by modulating neurotransmitter release and enhancing neuronal survival [28]. However, gabapentin primarily addresses symptomatic relief rather than the regenerative process. In contrast, the vitamin B complex not only improves functional recovery but also promotes myelination and reduces fibrosis, indicating a more comprehensive regenerative effect.

Nerve Growth Factor (NGF) has been studied for its potential to enhance nerve regeneration by promoting neuronal survival and axonal growth. While NGF shows promise, its clinical application is limited due to challenges in its delivery and potential side effects such as hyperalgesia [29,30]. Our study indicates that the vitamin B complex, when administered intraperitoneally, effectively enhances nerve regeneration with a safer profile.

Curcumin, a natural polyphenol, has anti-inflammatory and antioxidant properties that support nerve regeneration. Studies have shown that curcumin can reduce oxidative stress, inflammation, and apoptosis in nerve tissues, promoting functional recovery [31,32,33]. While curcumin’s effects are promising, the vitamin B complex offers additional benefits in maintaining myelin integrity and modulating neuroinflammation, as demonstrated in our study.

The vitamin B complex reduces pro-inflammatory cytokines and increases anti-inflammatory cytokines, facilitating the resolution of neuroinflammation. This shift from M1 to M2 macrophages promotes a favorable environment for nerve regeneration [24,34]. Vitamins B1, B6, and B12 are crucial for the synthesis and maintenance of myelin sheaths. Improved myelination observed in the treated groups indicates that the vitamin B complex supports the structural integrity of myelinated axons, which is essential for efficient nerve conduction [10,35,36]. Electrophysiological assessments revealed that vitamin B treatment significantly improved the latency, velocity, and peak-to-peak values in the nerve graft, transection, and crush injury models. These improvements suggest enhanced nerve conduction and functional recovery [35,36]. The findings from this study have significant clinical implications. The vitamin B complex could be considered a therapeutic option to enhance nerve regeneration and functional recovery in patients with peripheral nerve injuries. The safety profile and accessibility of vitamin B supplements make them a viable adjunct therapy in nerve repair strategies.

### Limitations of the Study

Despite the promising results, this study has limitations. The animal model used may not fully replicate the complexity of human peripheral nerve injuries. Additionally, the long-term effects of vitamin B treatment were not assessed. Future studies should focus on clinical trials to validate these findings in human subjects and explore the long-term benefits and potential side effects of vitamin B complex therapy.

## 5. Conclusions

In conclusion, vitamin B treatment significantly improves functional recovery and nerve regeneration in peripheral nerve injuries. The results from the walk test analysis, electrophysiological evaluation, and histopathological examination consistently indicate that vitamin B enhances nerve healing and reduces the pathological changes following nerve damage. These findings support the therapeutic potential of vitamin B in treating peripheral nerve injuries.

## Figures and Tables

**Table 1:** Comparison of electromyography (EMG) results of the groups.

	Latency (ms)	Velocity (m/s)	Peak-to-Peak (mV)
Control (Nerve graft)	3.5 ± 0.4	45 ± 5	0.8 ± 0.1
Vit B (Nerve graft)	3.0 ± 0.3 *	55 ± 6 *	1.2 ± 0.1 *
Control (Nerve transection)	4.0 ± 0.5	40 ± 4	0.5 ± 0.1
Vit B (Nerve transection)	3.3 ± 0.4 *	50 ± 5 *	1.0 ± 0.2 *
Control (Crush injury)	4.2 ± 0.6	38 ± 4	0.6 ± 0.1
Vit B (Crush injury)	3.6 ± 0.4 *	48 ± 5 *	1.1 ± 0.2 *

*: *p* < 0.05.

**Table 2:** Comparison of histopathological results between groups.

	Myelination	Fibrosis	Edema	Mast Cells
Control (nerve graft)	5.2 ± 0.6	1.4 ± 0.3	2.3 ± 0.4	3.1 ± 0.5
Vit B (nerve graft)	6.8 ± 0.7	1.0 ± 0.2	1.8 ± 0.3	2.5 ± 0.4
Control (nerve transection)	4.5 ± 0.5	2.0 ± 0.4	3.0 ± 0.5	3.6 ± 0.6
Vit B (nerve transection)	6.0 ± 0.6	1.2 ± 0.3	2.2 ± 0.4	2.8 ± 0.5
Control (crush injury)	4.8 ± 0.6	2.1 ± 0.4	2.9 ± 0.5	3.3 ± 0.5
Vit B (crush injury)	6.3 ± 0.7	1.3 ± 0.3	2.1 ± 0.4	2.9 ± 0.5
*p*-values				
Nerve graft	0.010	0.030	0.023	0.036
Nerve transection	0.022	0.041	0.034	0.045
Crush injury	0.013	0.034	0.027	0.038

## Data Availability

The original contributions presented in the study are included in the article, further inquiries can be directed to the corresponding authors.
